# Decrease in Behavioral Problems and Trauma Symptoms Among At-Risk Adopted Children Following Trauma-Informed Parent Training Intervention

**DOI:** 10.1007/s40653-015-0055-y

**Published:** 2015-08-06

**Authors:** Karyn B. Purvis, Erin Becker Razuri, Amanda R. Hiles Howard, Casey D. Call, Jamie Hurst DeLuna, Jordan S. Hall, David R. Cross

**Affiliations:** Institute of Child Development, Department of Psychology, Texas Christian University, Fort Worth, TX 76129 USA; Samford University, Birmingham, Alabama

**Keywords:** Trust-based relational intervention, Trauma-informed intervention, Attachment intervention, Out-of-home placement, Adopted children, Parent training, Child behavior, Trauma symptoms

## Abstract

Children who have experienced early adversities are at risk for behavioral problems and trauma symptoms. Using a two-group, pre-post intervention design, the current study evaluated the effectiveness of a parent training utilizing Trust-Based Relational Intervention, a trauma-informed, attachment-based intervention, in reducing behavioral problems and trauma symptoms in at-risk adopted children. Children of parents in the treatment group (*n* = 48) demonstrated significant decreases in behavioral problems on the Strengths and Difficulties Questionnaire and significant decreases in trauma symptoms on the Trauma Symptoms Checklist for Young Children after intervention. Scores for children in a matched-sample control group did not change. Findings suggest that Trust-Based Relational Intervention is effective at addressing many behavioral problems and trauma symptoms in children with histories of adversities.

Children who have experienced multiple and/or chronic adversities early in life, such as maltreatment, deprivation, and institutionalization, are at risk for poor developmental outcomes, including behavioral problems that can persist or get worse over time (Becker Weidman 2009; Kisiel et al. 2009b). Consistent with histories of early adversities, most children who have experienced out-of-home care, including institutional and foster placements, have experienced trauma (for a review of the literature, see Dovran et al. 2012). For example, children in out-of-home care are at an increased risk for abuse and neglect (Euser et al. 2013, 2014; Hobbs and Hobbs 1999; Johnson and Dole 1999). In addition to high rates of maltreatment, children in institutional or foster care have, by definition, experienced separation from or loss of the caregiver. These children are also at risk for having experienced other traumatic circumstances, including witnessing violence in the home or in armed conflict, exposure to natural disasters, and other chaotic or threatening environments (Hoksbergen and van Dijkum 2001).

Behavioral problems are one of the most salient challenges in caring for children with histories of early adversities. There is evidence of behavioral problems in post-institutionalized internationally adopted children (e.g., Gunnar et al. 2007), in at-risk domestically adopted children, and in mixed adoption samples (e.g., Groza and Ryan 2002; Kočovská et al. 2012). Findings include problems with internalizing behaviors (Fisher et al. 1997), externalizing behaviors (Hoksbergen et al. 2004; Merz and McCall 2010; Smith et al. 2000; Verhulst 2000), attention problems (Merz and McCall 2010; Groza 1999; Gunnar et al. 2007), thought problems (Groza 1999; Hoksbergen et al. 2004) and social problems (Gunnar et al. 2007; Hoksbergen et al. 2004). These behavioral problems can be extreme and/or pervasive (e.g., Groza and Ryan 2002; Gunnar et al. 2007; MacLean 2003; van der Vegt et al. 2009). The long-term trajectory for children who experience early adversity is often troublesome, with longitudinal research suggesting that at-risk adopted children exhibit increasingly more problem behaviors as they reach adolescence (Verhulst and Versluis-Den Bieman 1995; Verhulst 2000) and that early adversity increases the level of behavioral problems into adulthood (van der Vegt et al. 2009). These studies provide evidence that the long-term effects of early adversity are often pervasive and persistent even after removal from adverse circumstances.

Despite concern over the behavioral challenges in this vulnerable population, researchers note the paucity of empirical studies that assess trauma (Dovran et al. 2012; Hoksbergen and van Dijkum 2001). Research suggests that children in foster care in the United States exhibit symptoms of post-traumatic stress disorder (PTSD) at more than twice the rate of combat veterans (Pecora et al. 2009). Further, post-institutionalized children exhibit PTSD characteristics in line with survival-seeking behavior (Hoksbergen et al. 2003). However, the majority of studies with this population do not address trauma symptoms. For children with out-of-home placements, especially post-institutionalized, internationally adopted children, incomplete and/or unreliable information regarding pre-adoption circumstances (Gunnar et al. 2000) can make it difficult to confirm traumatic histories. In addition, there are questions as to whether the current diagnostic criteria for PTSD is appropriate for children, both because it is not developmentally sensitive and because it does not distinguish between acute trauma (exposure to a single overwhelming event such as a natural disaster) and chronic or multiple traumas (e.g., deprivation and maltreatment over years of institutional care) (Cook et al. 2005; van der Kolk 2005). The diagnosis of complex trauma, described by van der Kolk (2005) as “multiple, chronic and prolonged, developmentally adverse traumatic events, most often of an interpersonal nature…and early-life onset” (p. 402) captures the extended exposure to early adversities often suffered by children from institutional or foster care. As complex trauma becomes a more widely-recognized framework for evaluating vulnerable children, early evidence provides a link between behavioral challenges and trauma (Kisiel et al. 2009a, b, 2014). For example, Milot and colleagues (Milot et al. 2010) suggest that the association between behavioral problems and maltreatment might be mediated by trauma symptoms. However, more empirical support is necessary to link the established behavioral problems seen in at-risk adopted children to trauma.

## Trust-Based Relational Intervention as Trauma-Informed Care

The number of adoptive families potentially impacted by behavioral challenges and trauma symptoms in children with histories of early adversities is substantial. Over 240,000 children have been adopted into the United States from other countries since 1999 (U.S. Department of State), the majority of whom were institutionalized before adoption and at risk for multiple traumas, including maltreatment and deprivation (Gunnar et al. 2007). In addition, 50,000 children are adopted within the United States after child welfare agency involvement every year (U.S. Department of Health and Human Services 2011). Effective, trauma-informed interventions are necessary to support the large number of adoptive families caring for children whose histories of early adversity put them at risk for a range of developmental deficits (Ko et al. 2008). In their 25-year review of empirical research of trauma in children with out-of-home placements, Dovran and colleagues (2012) note that only one study could be found that focused on evidence-based treatment and trauma-informed care (Weiner et al. 2009). Successful interventions must address the persistent and extreme behavioral issues often exhibited by at-risk adopted children, but also acknowledge underlying trauma that is often overlooked.

Based on a review of the literature, Howard Bath has proposed three pillars of trauma-informed care that are necessary for helping children heal from complex trauma: Felt-Safety, Self-Regulation, and Connection (Bath 2008). These appear to be the fundamental elements of established trauma-informed interventions, including the Attachment, Self-Regulation, and Competency Model (ARC; Arvidson et al. 2011), Attachment and Biobehavioral Catch-Up (Dozier et al. 2008) and Circle of Security (Hoffman et al. 2006). From an attachment theory perspective, it is notable that these trauma-informed interventions all recognize the importance of intervention within the context of the caregiving relationship. Indeed, the third pillar of trauma-informed care, connection, is the foundation for the first two pillars. Felt-safety is derived from trusting relationships (Eisenberger et al. 2011), an idea that is central to attachment theory (see Bretherton 1992). Further, self-regulation is also derived from sensitive, trusting relationships, through countless regulatory and attuned caregiver-child interactions (Schore 2001). Researchers agree that for children suffering multiple or chronic early adversities, interventions targeting attachment are necessary (Cook et al. 2005; Dozier et al. 2002; Hawk and McCall 2010; MacLean 2003). Although behavioral change is a goal of intervention, the ultimate objective is to enhance the relationship between the child and caregiver, what Dozier and colleagues call the “critical transducer of change” (Dozier et al. 2002, p. 856). Improving the quality of the child-caregiver relationship improves physical, mental, social, and emotional development in at-risk children (Dozier et al. 2002; St. Petersburg-USA Orphanage Research Team 2008). A high quality caregiver-child relationship, such as that found in a secure attachment dyad, can buffer against the harmful effects of early adverse experiences, including poor behavioral outcomes (Audet and Le Mare 2010; Colonnesia et al. 2012; Kriebel and Wentzel 2011; McGoron et al. 2012).

TBRI is a trauma-informed intervention grounded in attachment theory that seeks to improve outcomes for vulnerable children by (1) helping caregivers see the needs of children who have experienced relational trauma and (2) helping caregivers do what is necessary to meet those needs. For intervention to be most effective the underlying trauma experienced by children with histories of maltreatment and deprivation must be addressed. In line with the three pillars of trauma-informed care, TBRI consists of three sets of principles that facilitate felt-safety, self-regulation, and connection: Empowering Principles, Connecting Principles, and Correcting Principles. Each set of principles has two associated sets of strategies.

The two sets of strategies associated with Empowering Principles are (1) Ecological Strategies, such as recognizing and managing transitions and establishing rituals that structure and connect and (2) Physiological Strategies, such as providing regular physical activity and sensory experiences and meeting nutritional and hydration needs. The basic idea of the Empowering Principles is that by attending to these principles and strategies, caregivers can enhance a child’s capacity for self-regulation, decrease the likelihood of negative and disruptive incidents, and increase the likelihood of successful “connecting and correcting.”

The sets of strategies associated with Connecting Principles are (1) Mindful Awareness, such as an awareness of the child, the self, and the environment and (2) Engagement Strategies, such as valuing eye contact, playful interaction, and healthy touch. The Connecting Principles are based on attachment theory and research (see Cassidy and Shaver 2008; Siegel 2012) and are the “heart and soul” of TBRI. The Connecting Principles are not only important in their own right, as essential mechanisms for building trusting relationships, but are also the engine that makes both the Empowering and the Correcting Principles work in practice.

The strategies associated with Correcting Strategies consist of (1) Proactive Strategies, such as teaching Life Value Terms and Behavioral Scripts during playful interactions and (2) Responsive Strategies, such as using the IDEAL Response and Levels of Response to respond to challenging behavior. The Correcting Principles are used to deliberately shape behavior, but will only be effective to the extent that their practice is based on a firm foundation of Empowering and Connecting. A more detailed explanation of TBRI and how the principles are applied can be found in previous publications (e.g., Purvis et al. 2013a, b).

## The Current Study

TBRI has been used in a number of settings to effect change, including intensive home programs (McKenzie et al. 2014), residential treatment centers (Purvis et al. 2012, 2014), and schools (Parris et al. 2014), but the current study is the first to use a randomized sample, pre-post design with a control group to evaluate the effectiveness of the intervention. Consistent with research demonstrating the efficacy of trauma-informed, attachment-based interventions on behavioral outcomes for at-risk children (Dozier et al. 2002) and with research suggesting that trauma symptoms account for the relationship between early adversity and behavioral problems (Milot et al. 2010), it is expected that behavioral problems and trauma symptoms will decrease for at-risk adopted children whose parents participate in a TBRI trauma-informed parent-training program.

## Method

### Participants

Participants consisted of 96 adoptive parents who responded to a recruitment notice for a study for parents interested in learning about the basic relationship and developmental needs of adopted children with histories of early adversities and practical strategies to improve outcomes for these children. Recruitment notices were posted on the university website, distributed by email through child welfare professionals across the United States, and emailed to parents on the research institute’s distribution list. Eligible participants included parents of children who were domestically or internationally adopted, were between the ages of 5 and 12 at the beginning of the study, and had resided in the adoptive home for at least 1 year. In addition, parents or other immediate family members could not have participated in previous training or research studies hosted by the research institute. Parent training was offered free of charge.

Of the 492 eligible participants who responded to the recruitment notice, 167 indicated that they were available to travel to the university (located in a large metropolitan area in the southern United States) to attend 4 days of on-site parent training. Of these 167 potential participants, 85 were randomly assigned to attend parent training (treatment group). The remaining 82, along with the 325 eligible participants who indicated that they were interested in receiving training but were unavailable to attend on-site training, were randomly assigned to either an online treatment group (*n* = 205, reported elsewhere) or a control group (*n* = 202). Participants in the control group were offered online training after the conclusion of the study. Reported here are results for the 48 participants in the treatment group and 48 participants in a matched sample control group who had complete data through the final round of data collection (attrition rate = 29% for treatment group, 14% for control group). Control group participants were matched to treatment group on child sex, age, adoption type (domestic vs. international), and age at adoption (within 9 months). Means and standard deviations for continuous descriptive variables by group (control vs. treatment) for the child can be found in Table 1. Further, frequencies and percentage for categorical descriptive variables by group for the child can be found in Table 2. Means and standard deviations for continuous descriptive variables by group for the primary caregiver can be found in Table 3. Further, frequencies and percentage for categorical descriptive variables by group for the primary caregiver can be found in Table 4. As can be seen in Tables 1, 2, 3 and 4, the treatment and control groups did not differ significantly on any descriptive variables.

**Table 1:** Means and standard deviations for continuous descriptive variables by group for the child

	Mean	SD	*F*	*p*
Current age in years			.00	1.00
Treatment	7.88	2.06		
Control	7.88	2.01		
Age at adoption in months			.38	.54
Treatment	33.69	31.76		
Control	37.70	32.29		
Length of time in home in months			.16	.70
Treatment	66.79	35.41		
Control	64.19	29.12		

*p < .05, **p < .01

**Table 2:** Frequencies and percentage for categorical descriptive variables by group for the child

	Treatment		Control			
	n	%	n	%	*χ* ^*2*^	*p*
Child sex					.00	1.00
Male	30	62.5%	30	62.5%		
Female	18	37.5%	18	37.5%		
Child ethnicity					7.98	.16
Asian	9	18.8%	12	25.0%		
Black/African American	13	27.1%	16	33.3%		
Hispanic/Latino	1	2.1%	4	8.3%		
White/Caucasian	20	41.7%	16	33.3%		
Native American	1	2.1%	0	0.0%		
Other	4	8.3%	0	0.0%		
Adoption type					.00	1.00
Domestic	18	37.5%	18	37.5%		
International	30	62.5%	30	62.5%		
Neglect					.22	.64
Yes	35	72.9%	37	77.1%		
No	13	27.1%	11	22.9%		
Physical abuse					1.69	.19
Yes	13	27.1%	19	39.6%		
No	35	72.9%	29	60.4%		
Sexual abuse					.00	1.00
Yes	7	14.6%	7	14.6%		
No	41	85.4%	41	85.4%		

*p < .05, **p < .01

**Table 3:** Means and standard deviations for continuous descriptive variables by group for the primary caregiver

	Mean	SD	*F*	*p*
Parent current age			2.74	.10
Treatment	43.79	6.01		
Control	41.90	5.19		
Number of children in home			.06	.82
Treatment	3.02	1.21		
Control	3.08	1.40		

* p < .05, **p < .01

**Table 4:** Frequencies and percentage for categorical descriptive variables by group for the primary caregiver

	Treatment		Control			
	n	%	n	%	*χ* ^*2*^	*p*
Parent sex					3.10	.08
Male	3	6.3%	0	0.0%		
Female	45	93.8%	48	100.0%		
Parent ethnicity					1.01	.32
Hispanic/Latino	1	2.1%	0	0.0%		
White/Caucasian	47	97.9%	48	100.0%		
Marital status					1.43	.49
Single	3	6.3%	1	2.1%		
Married	43	89.6%	46	95.8%		
Divorced	2	4.2%	1	2.1%		
Parent education					3.06	.38
High school	1	2.1%	0	0.0%		
Some college	6	12.5%	6	12.5%		
College degree	21	43.8%	28	58.3%		
Advanced degree	20	41.7%	14	29.2%		
Income					4.91	.43
$25,000–$34,999	2	4.2%	0	0.0%		
$35,000–49,999	4	8.3%	2	4.2%		
$50,000–$74,999	6	12.5%	12	25.0%		
$75,000–$99,999	9	18.8%	10	20.8%		
$100,000–$150,000	15	31.3%	13	27.1%		
$150,000+	12	25.0%	11	22.9%		
TBRI exposure					3.38	.07
Yes	30	62.5%	21	43.8%		
No	18	37.5%	27	56.3%		

* p < .05, **p < .01

### Procedure

Prior to data collection, ethical approval was obtained from the University’s Institutional Review Board. All participants provided informed consent before participating in research. All participants participated in an online pretest approximately 2 weeks before intervention began, as well as an online posttest approximately 2 weeks after intervention ended. Primary caregivers completed the Strengths and Difficulties Questionnaire (SDQ; Goodman 2001) and Trauma Symptoms Checklist for Young Children (TSCYC; Briere 2001) at both pre- and posttest. Psychometric properties of the questionnaires for both pre and post treatment can be found in Table 5. In addition, participants provided background information, including demographics and the child’s pre-adoption history, at pretest. The questionnaires making up the pre- and posttest assessments were presented in random order at each administration.

**Table 5:** Psychometric properties of the questionnaires pre and post treatment

	α Pre-treatment	α Post-treatment
SDQ		
Emotional problems	.66	.63
Conduct problems	.63	.65
Hyperactivity	.73	.73
Peer problems	.57	.67
Prosocial behavior	.69	.72
Total problems	.66	.68
TSCYC		
Anxiety	.83	.87
Depression	.85	.82
Anger/Aggression	.93	.91
Posttraumatic stress - intrusion	.86	.89
Posttraumatic stress - avoidance	.80	.79
Posttraumatic stress - arousal	.83	.83
Posttraumatic stress - total	.83	.86
Dissociation	.91	.93
Sexual concerns	.82	.80

### Assessments

#### Strengths & Difficulties Questionnaire

The SDQ is a 25-item measure of behavior for children age 3 to 16 years old that can be completed by parents, teachers, or adolescents. The SDQ assesses four domains of behavioral problems: Emotional Symptoms, Conduct Problems, Hyperactivity/Inattention, and Peer Problems. Added together, scores from these four subscales give a Total Difficulties score. The SDQ also assesses Prosocial Behavior. Each item is rated on a 3-point scale (0 = not true to 2 = certainly true). The SDQ has good reliability and validity (Goodman 2001).

#### Trauma Symptom Checklist for Young Children

The TSCYC is a 90-item caregiver-report measure of acute and chronic posttraumatic symptomology in children age 3 to 12 years old. The TSCYC yields eight clinical subscales: Anxiety, Depression, Anger/Aggression, Post-traumatic Stress-Intrusion, Post-traumatic Stress-Avoidance, Post-traumatic Stress-Arousal, Post-traumatic Stress-Total, Sexual Concerns, and Dissociation. The TSCYC also contains two validity scales: Response Level, which indicates a tendency for reporters to over-respond, and Atypical Response, which indicates a tendency to under-respond. Each item is rated on a 4-point scale (from 1 = not at all to 4 = very often). The TSCYC clinical scales have good reliability (average scale *alpha* of .87; Briere et al. 2001) and good convergent validity with other parent-report measures (Trauma Symptom Checklist for Children, Child Behavior Checklist, and the UCLA PTSD Index-Parent Form; Wherry et al. 2008).

### Intervention Protocol

Participants in the treatment group attended a 4-day TBRI parent training (6 h per day) designed to teach strategies and skills intended to improve behavioral outcomes for children with histories of complex trauma. The training consisted of 1 day each of TBRI Introduction & Overview, Empowering, Connecting, and Correcting Principles. The training utilized standardized presentations, presenter manuals, and participant workbooks routinely used in TBRI workshops with various audiences interested in creating changes for children with early adverse histories, including audiences of child welfare professionals, teachers, and adoptive and foster parents. Each day of training consisted of interactive lectures with small and large group discussions, application activities such as role-plays and therapeutic groups, and video clips used to demonstrate principles and strategies. Trainers each had approximately 2 years experience using the standardized presentations, manuals, and workbooks.

## Results

The outcomes of the parent training intervention on the SDQ and TSCYC subscales were examined by repeated measures Multivariate Analysis of Covariance (MANCOVAs) with time (pre and post) as the within subjects factor, group (treatment and control) as the between subjects factor, and child’s sex and current age as the covariates. Tables 5 and 6 present the descriptive statistics and the MANCOVA results for the SDQ and TSCYC subscales respectively. Tables include *F-*values for simple and interaction effects as well as related effect sizes (η_p_^2^).

**Table 6:** Results for the strengths and difficulties questionnaire by group: descriptives (means and standard deviations) and generalized linear model results (F-values (partial η^2^)

	Treatment		Control				
	Mean	SD	Mean	SD	Time (η_p_^2^)	Group (η_p_^2^)	Interaction (η_p_^2^)
Emotional problems					.48 (.01)	.74 (.01)	3.99 (.04)*
Pre	4.10	2.38	4.02	2.21			
Post	3.25	2.33	4.06	2.45			
Conduct problems					.08 (.00)	.07 (.00)	3.95 (.04)*
Pre	5.17	2.37	4.96	2.40			
Post	4.58	2.30	5.04	2.67			
Hyperactivity/Inattention					.07 (.00)	.02 (.00)	9.07 (.09)**
Pre	6.08	1.85	5.73	2.08			
Post	5.48	2.03	5.94	1.74			
Peer problems					5.69 (.06)*	1.53 (.02)	.01 (.00)
Pre	3.54	2.43	2.98	2.15			
Post	3.58	2.50	3.04	2.00			
Prosocial behavior					2.18 (.02)	.68 (.00)	4.33 (.05)*
Pre	4.90	2.04	5.06	2.06			
Post	6.21	1.87	5.71	2.41			
Total difficulties					1.38 (.02)	.00 (.00)	8.97 (.09)**
Pre	18.90	6.83	17.69	5.46			
Post	16.90	6.64	18.08	5.87			

* p < .05, **p < .01

As can be seen in Table 6, results revealed significant interaction effects for time and group for four of the five SDQ subscales and Total Difficulties. Caregiver reports of the child’s Emotional Problems, Conduct Problems, Hyperactivity/Inattention, and Total Difficulties were significantly lower at posttest than at pretest for the treatment group, but did not significantly change over time for the control group. Further, caregiver reports of the child’s Prosocial Behavior were significantly higher following intervention for the treatment group, but did not change over time for the control group. Results also revealed a significant main effect for time on the SDQ Peer Problems subscale. On average, parents reported more peer-related problems at posttest (*M*_pre_ = 3.26, *SD* = 2.29; *M*_post_ = 3.31, *SD* = 2.25). Results also revealed significant interaction between time and child’s current age for the Emotional Problems (*F* = 4.51, *p* < .05*,* η_p_^2^ = .05), Peer Problems (*F* = 7.65, *p* < .01*,* η_p_^2^ = .08), and Total Difficulties (*F* = 6.05, *p* < .05*,* η_p_^2^ = .06). Parents reported that older children had a greater decrease in problem behavior than younger children from pretest to posttest regardless of group.

As can be seen in Table 7, results revealed significant interaction effects for time and group for *T* scores on four of the nine TSCYC scales. Caregiver reports of the child’s Anxiety, Depression, and Post-traumatic stress-Arousal significantly decreased from pretest to posttest for the treatment group, but did not change significantly for the control group. Further, results showed a significant main effect for time and a significant interaction effect for time and group for the Anger/Aggression scale. On average, parents reported lower levels of Anger/Aggression at posttest (*M*_pre_ = 68.06, *SD* = 17.60; *M*_post_ = 65.53, *SD* = 17.87). Though both groups changed from pretest to posttest, the treatment group had a significantly larger decrease in Anger/Aggression scores at posttest (*M*_pre_ = 70.94, *SD* = 18.44; *M*_post_ = 66.04, *SD* = 18.43) than the control group (*M*_pre_ = 65.17, *SD* = 16.75; *M*_post_ = 65.02, *SD* = 17.30). No interaction effects were found on the TSCYC scales between the child’s sex, current age, time, and group.

**Table 7:** Results for the trauma symptoms checklist by group: descriptives (means and standard deviations) and generalized linear model results (F-values (partial η^2^)

	Treatment	Control					
	Mean	SD	Mean	SD	Time (η_p_^2^)	Group (η_p_^2^)	Interaction (η_p_^2^)
Anxiety					.45 (.01)	2.16 (.02)	3.98 (.04)*
Pre	59.46	11.87	61.23	15.09			
Post	55.27	11.31	60.50	13.08			
Depression					.12 (.00)	3.89 (.04)*	3.83 (.04)*
Pre	56.81	12.09	59.27	12.21			
Post	53.00	11.69	59.35	12.19			
Anger/Aggression					6.95 (.07)**	1.09 (.01)	6.02 (.06)*
Pre	70.94	18.44	65.17	16.75			
Post	66.04	18.43	65.02	17.30			
PTS intrusion					.59 (.01)	.01 (.00)	1.58 (.02)
Pre	53.63	12.79	55.02	12.29			
Post	55.08	13.60	54.19	10.58			
PTS avoidance					.18 (.00)	1.96 (.02)	2.24 (.02)
Pre	62.29	14.94	56.90	12.44			
Post	60.69	15.11	58.52	14.75			
PTS arousal					.27 (.00)	.11 (.00)	9.47 (.09)**
Pre	68.50	17.81	64.63	15.29			
Post	64.29	15.70	66.10	17.08			
PTS total					.65 (.01)	.43 (.01)	2.63 (.03)
Pre	61.47	11.97	58.85	11.32			
Post	60.02	12.51	59.60	12.20			
Dissociation					1.89 (.02)	1.78 (.02)	.16 (.00)
Pre	55.98	12.24	59.15	13.94			
Post	56.58	15.37	60.48	14.26			
Sexual concerns					.20 (.00)	1.36 (.02)	.10 (.00)
Pre	51.77	9.75	49.63	7.30			
Post	53.10	13.84	50.58	9.92			

*PTS* post-traumatic stress

* p < .05, **p < .01

## Discussion

The current study reports on an intervention that is effective at reducing many behavioral problems and trauma symptoms among vulnerable children. In terms of behavior, parents in the treatment group reported significantly lower scores for Conduct Problems, Hyperactivity/Inattention, Emotional Problems, and Total Problems and significantly higher scores for Prosocial Behavior on the SDQ after intervention, while behaviors in the control group remained unchanged. In terms of symptoms of trauma, parents in the treatment group reported that their children had significantly lower Anxiety, Depression, Anger/Aggression, and Post-Traumatic Stress-Arousal on the TSCYC after intervention, while trauma symptoms in the control group remained unchanged.

In line with the principles of trauma-informed care, the core values of TBRI are felt-safety, self-regulation, and connection. Although previous research suggests that focusing on these core values will result in behavioral change, this is the first TBRI study to empirically assess change in trauma symptoms. Not surprisingly given their early adverse histories, children in the current sample are at risk for symptoms in line with a Post-Traumatic Stress diagnosis, including arousal, intrusion, and avoidance. That a 4-day parent training could effectively decrease trauma symptomology speaks to the importance of considering trauma in the context of the caregiver-child relationship. Indeed, although constructs such as “felt-safety” are difficult to capture in behavioral terms, decreases in arousal and anxiety suggest that children feel safe.

These findings are encouraging for the multitude of families seeking help for the behavioral challenges exhibited by their at-risk adopted children every day and are also important for researchers who seek to identify developmental domains that should be targeted for interventions. Notably, the improvements evident after intervention were not limited to one domain, but appeared in various domains that are addressed by the intervention. For example, conduct problems are addressed proactively with TBRI strategies, such as role-playing to practice “using your words.” Hyperactivity is addressed with strategies focused on sensory stimulation. Emotional problems are addressed in activities such as creating a clay model representing something the child is afraid of and then smashing the model. Although one goal of the intervention is behavioral change, it is most effective in the context of the safety, security, and sensitivity of a healthy attachment relationship. Thus, although the intervention utilizes both Proactive and Responsive strategies for encouraging pro-social behavior and discouraging maladaptive behaviors, scripts such as “use your words” or “gentle and kind” carry no weight if not used appropriately by safe, nurturing adults who try to build connection even when correcting behavior. Consistent with this perspective, it is important that the intervention target caregivers, with the intention that positive outcomes will be more long-lasting if the caregiver is the medium for change.

### Limitations and Future Research

Although promising, there are limitations to the current sample. Participants consisted of volunteers who were interested in learning strategies for improving outcomes for their adopted children. They had to have the means and time to travel to the training and the ability to be available for 4 days; therefore, this sample might not represent the population as a whole. Another possible limitation is the use of parent-report measures of child behavior. It is possible that problem behaviors decreased in the treatment group after intervention because parents wanted behaviors to decrease. However, the post-intervention improvements in behavior and trauma symptoms are supported by post-intervention improvements found in other studies utilizing different measures for the same intervention principles, including changes in neurochemistry (Cross et al. 2011; Purvis et al. 20111), attachment behavior (Purvis et al. 2013a, b), and social/emotional skills (Purvis et al. 2011). In addition, other studies have found high validity between parent reports of behavior and other behavioral measures, including teacher report (Kriebel and Wentzel 2011; Miller et al. 2009) and self-report ( Gagnon-Oosterwaal et al. 2012). As is common in studies of adopted children with early adverse histories, especially post-institutionalized children, there was no direct measure of pre-adoption experiences.

The current study reports on short-term improvements in behavior and trauma symptoms. Although encouraging, future research should assess long-term follow-up of outcomes following intervention to examine whether behavioral improvements last. If decreases in child behavioral problems and trauma symptoms are the result of changes to parent-child interactions stemming from TBRI parent training, then a central question for future research is to what extent parents continue to use the skills and strategies learned in training.

On-site TBRI training conducted face-to-face with TBRI-trained staff is a valuable training model: it allows for a dialogue between participants and trainers during which questions can be answered immediately, skills can be practiced with feedback, participants have a devoted time and space in which to learn and have the context of a supportive environment from trainers and other participants. However, on-site training also has its caveats: it limits the number of individuals who can attend training and it requires a lot of time and resources on the part of the training staff. Indeed, among the participants who were randomly assigned to the treatment group (who had all indicated that they were available to attend training on location during the training dates), the main reason participants withdrew from the study prior to intervention was difficulty making travel arrangements (e.g., unable to find child care, take time off of work, or afford travel expenses). The majority of participants who withdrew from the study expressed regret or frustration at not attending training. Future research will evaluate alternatives to on-site trainings led by research institute staff. A promising alternative is web-based training, which aims to provide the same content but allows participants to progress through training at their own speed on their own timeframe, no travel required. Future research will report on the efficacy of a pilot study of TBRI Online.

Children who have experienced chronic or multiple early adversities can bear the scars of their trauma long after removal from harsh environments. The effects can manifest in a number of ways, including behavioral problems and trauma symptoms. However, results of this study suggest that trauma-informed TBRI training can improve child outcomes and give families hope.
